# Graft-versus-host disease complicated with small bowel obstruction in children: A case report

**DOI:** 10.3389/fonc.2022.1002333

**Published:** 2022-09-08

**Authors:** Yizhong Wang, Jiangbin Liu, Bingxin Jiang, Chenling Yuan, Licai Chen, Ting Zhang, Zhibao Lv

**Affiliations:** ^1^ Department of Gastroenterology, Hepatology and Nutrition, Shanghai Children’s Hospital, School of Medicine, Shanghai Jiao Tong University, Shanghai, China; ^2^ Gut Microbiota and Metabolic Research Center, Institute of Pediatric Infection, Immunity and Critical Care Medicine, School of Medicine, Shanghai Jiao Tong University, Shanghai, China; ^3^ Department of General Surgery, Shanghai Children’s Hospital, School of Medicine, Shanghai Jiao Tong University, Shanghai, China

**Keywords:** graft-versus-host disease, hematopoietic cell transplantation, bowel obstruction, child, leukemia

## Abstract

Graft-versus-host disease (GvHD) is a severe complication following hematopoietic cell transplantation (HCT). The clinical manifestations of GvHD can affect multiple systems. Although gastrointestinal (GI) GvHD is common, GI obstruction complications are rare. Here, we present a case of GI-GvHD after HCT for acute myeloid leukemia (AML) in a young girl from China. The patient suffered from watery diarrhea, which progressed to bloody diarrhea 40 days after HCT. She experienced prolonged and repeated mucous or bloody stool after the withdrawal of cyclosporine and the gradual reduction in methylprednisolone. The plain abdominal radiography and computed tomographic (CT) scan showed apparent bowel wall thickening and intestinal stenosis 10 months after HCT. Finally, the patient underwent surgery to remove the small intestinal stenosis at the age of 26 months. The patient recovered with the help of appropriate medical therapies and nutritional support during hospitalization. She remained stable, and there was no recurrence of GI symptoms 16 months after the surgery. In summary, surgery may be an optimal treatment for GvHD patients with persistent bowel obstruction and failure of appropriate immunosuppressive therapies.

## Introduction

Graft-versus-host disease (GvHD) following hematopoietic cell transplantation (HCT) is a serious complication that can be life threatening to recipients. The occurrence of GvHD is attributable to the presence of immunocompetent T lymphocytes in the graft attacking the immunodeficient recipient tissue, ultimately causing host damage (1). Emerging evidence has revealed that multiple factors, including proinflammatory intracellular signaling activation, intestinal tissue regeneration defects, impaired antipathogen immunity, and dysbiosis of the gut microbiota, play critical roles in the pathogenesis of GvHD (2). The clinical manifestations of GvHD involve multiple systems, such as the skin, gastrointestinal (GI) tract, lungs, hepatobiliary system, musculoskeletal system, kidneys, eyes, and hematopoietic system (3). GI-GvHD, especially in the small intestine, is a leading cause of HCT-related morbidity and mortality in recipients (4). The clinical management of GvHD is complex and not standardized worldwide due to the limited results from well-designed, large-scale clinical studies. Immunosuppressive therapy is the most common intervention for the prophylaxis and treatment of GvHD, particularly acute GvHD that occurs in the short term after HCT (5). Recently, studies reported rare pediatric cases of small bowel obstruction caused by severe chronic intestinal GvHD that were managed efficiently with surgical intervention (6, 7). Here, we report a 26-month-old girl affected with small intestinal obstruction caused by chronic GI-GvHD after allogeneic HCT (allo-HCT) for acute myeloid leukemia (AML) who was treated successfully by surgery in China.

## Case presentation

**Figure 1:**
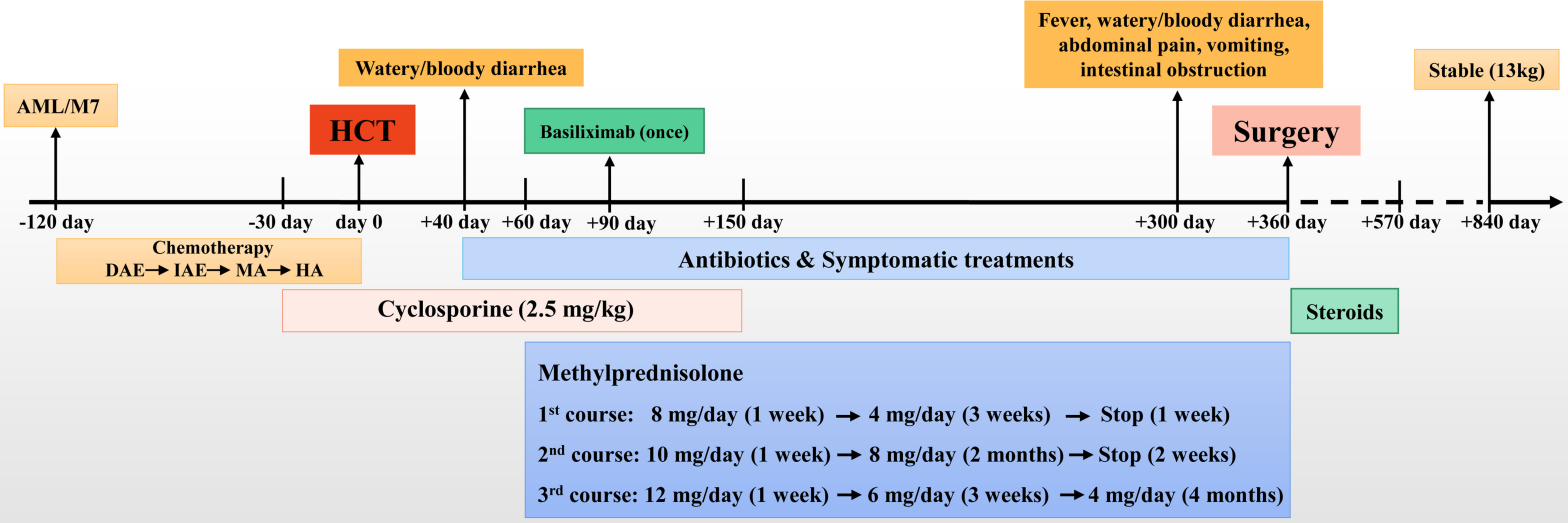
The timeline of the treatments. AML, acute myeloid leukemia; HCT, hematopoietic cell transplantation.

The patient was a girl diagnosed with AML and monosomy 7 at 10 months old (**Figure 1**). She received allo-HCT from a fully human leukocyte antigen (HLA)-matched unrelated donor (a 30-year-old female volunteer from the China Marrow Donor Program, 10/10 high resolution) after 4 months of chemotherapies with a standard protocol of DAE (DNR, Ara-C, and VP-16), IAE (IDA, Ara-C, and VP-16), MA (mito and Ara-C), and HA (Ara-C) based on the AML-SCMC-2009 protocol (8). Sustained cyclosporine (2.5 mg/kg) was used as prophylaxis for GvHD from 1 month before HCT until 5 months after HCT (**Figure 1**). She had no medical history of GI problems or GI allergies before HCT, and the patient was not complicated with GI manifestations during chemotherapy. On day 40 after HCT, the patient started to suffer from watery diarrhea, followed by bloody diarrhea, and was readmitted to our hospital. She had no fever, abdominal pain, cramps, or maculopapular rash. Abdominal ultrasonography revealed partial small-bowel wall thickening and lumen expansion. The colonoscopy was normal, and biopsy of the sigmoid colon showed chronic inflammation of the mucosa. Empiric antibiotic therapies (tigecycline and levofloxacin, followed by meropenem, vancomycin, and voriconazole) were first applied to treat possible intestinal infections. However, the diarrhea did not improve after 3 weeks of antibiotic therapy, and acute GI-GvHD was suspected. Then, the patient was treated with cyclosporine, basiliximab (once), and methylprednisolone, allowing a transient improvement of the GI symptoms (yellow loose stool, one to two times per day). During the withdrawal of cyclosporine and the gradual reduction in methylprednisolone (three courses of treatment, **Figure 1**), the patient experienced prolonged and repeated GI symptoms of mucous or bloody stool, along with recurrent hypoalbuminemia (albumin, 19.7–30.6 g/L). Albumin, gamma globulin, and frozen plasma were given to treat her hypoalbuminemia monthly.

**Figure 2:**
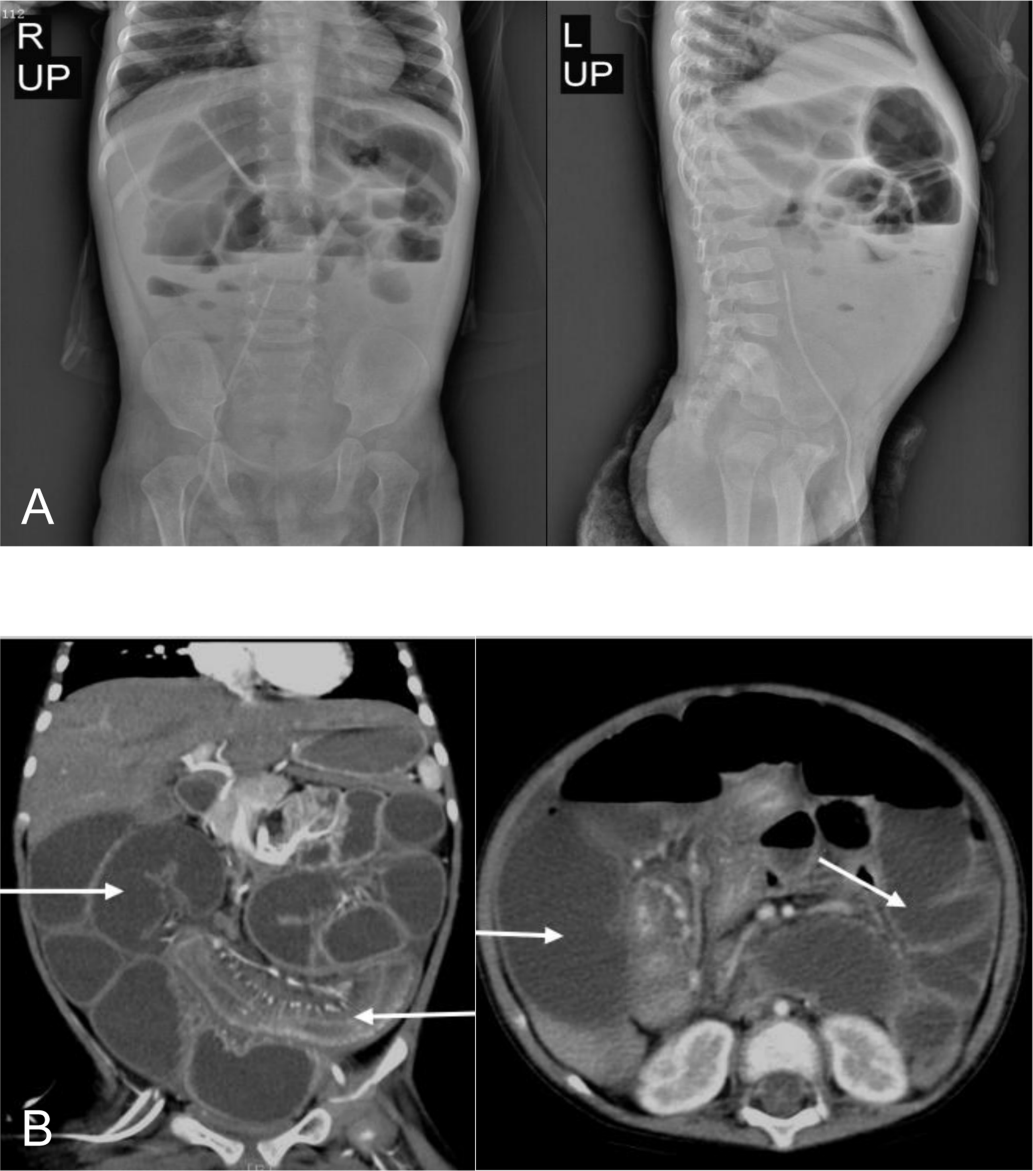
Bowel dilation, bowel wall thickening, and stenosis in the patient. **(A)** Abdominal radiograph showed bowel dilation with a gas−liquid level. **(B)** CT scan showed bowel dilation, thickening of the bowel wall, and small bowel stenosis.

**Figure 3:**
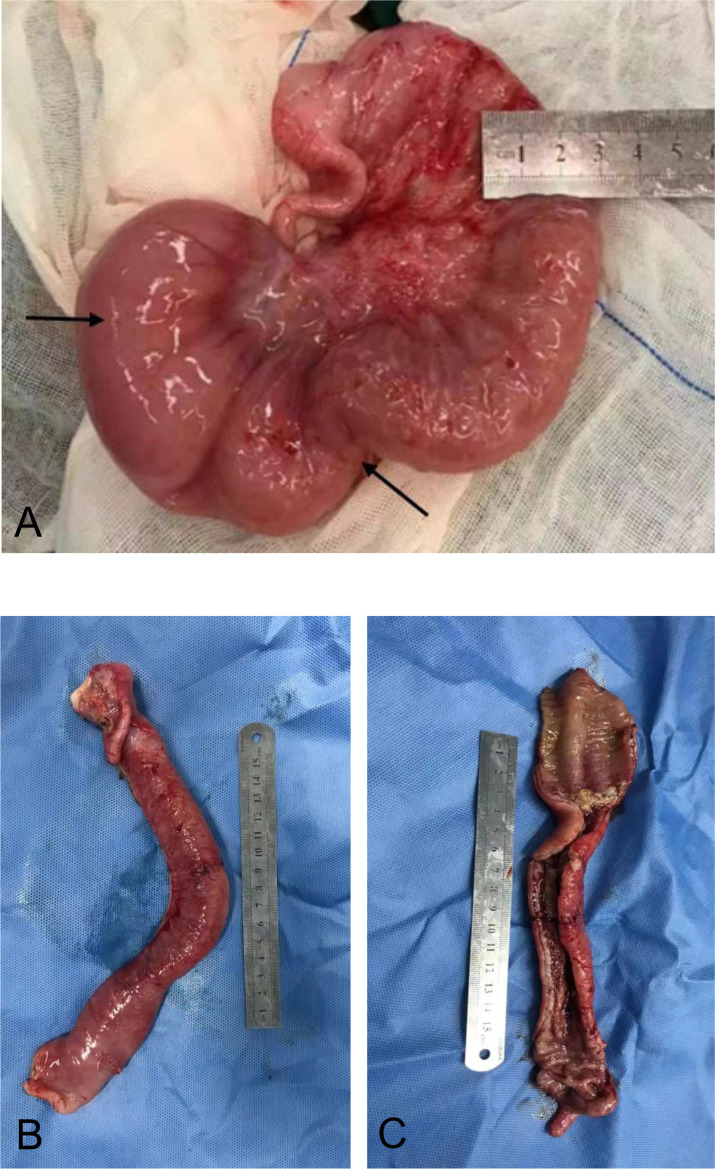
Surgical resection of the affected small intestine. **(A)** Small intestine wall thickening, edema, and stenosis. **(B)** Stiffness of the affected small intestine. **(C)** Reduction in the mucosal folds.

**Figure 4:**
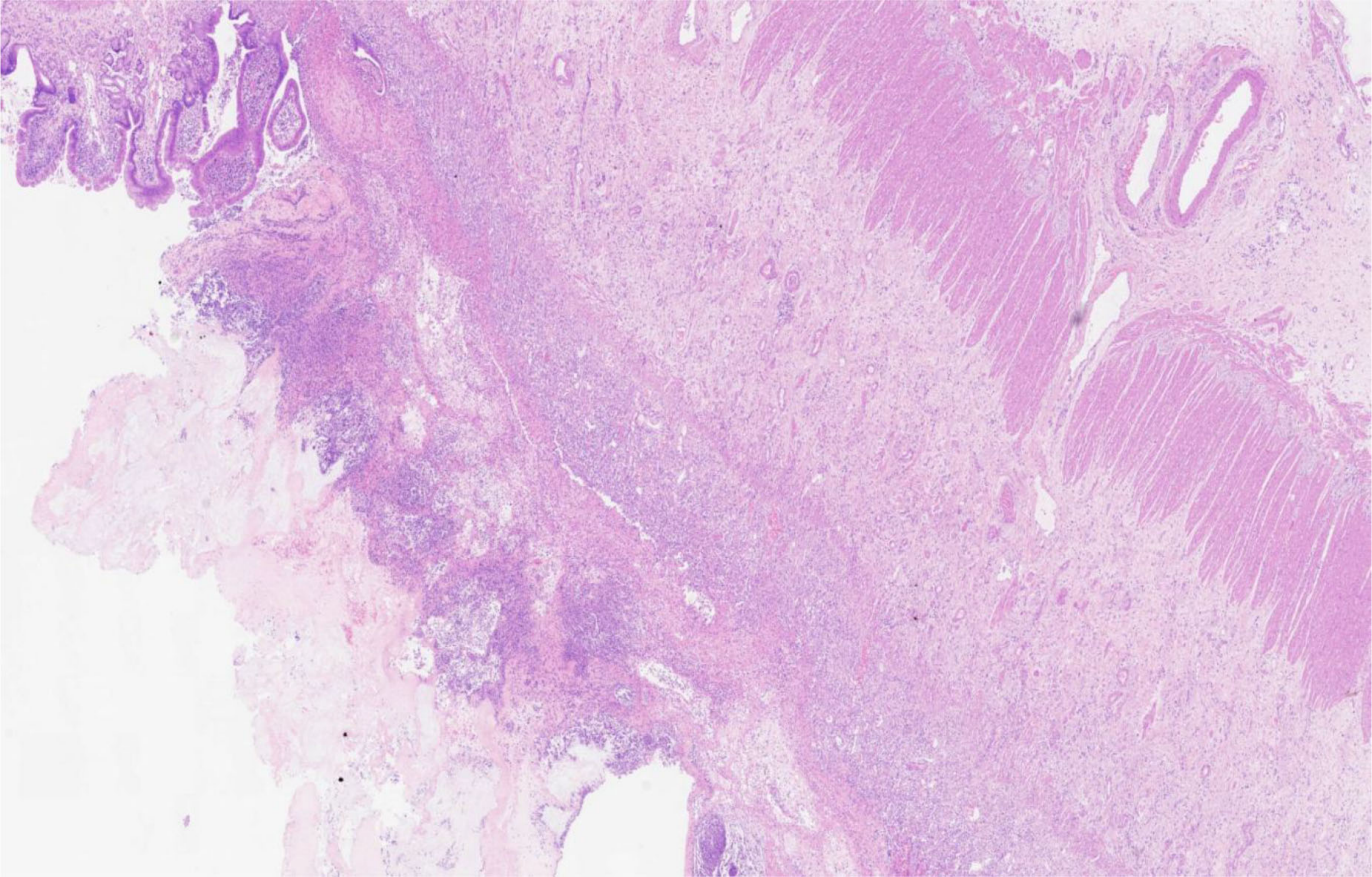
Histological examination showed crypt loss, marked inflammation, and associated architectural distortion (hematoxylin and eosin, 200×).

Ten months after HCT, fever and abdominal pain followed by vomiting and diarrhea occurred. Plain abdominal radiography and abdominal ultrasound suggested intestinal obstruction. The pathogens of Epstein–Barr virus (EBV), TORCH, and cytomegalovirus (CMV) were all negative. Other causes of bloody diarrhea, including *Salmonella* and *Clostridium difficile*, were not detected in her feces. No sign of clinical manifestations was observed in other organs, including the skin, lungs, liver, kidneys, and eyes. The patient was treated with conservative medical treatments, including fasting, parenteral nutrition, and antibiotics (ceftriaxone, meropenem, and itraconazole), but the symptoms did not improve. Repeat plain abdominal radiography revealed bowel dilation exacerbation with an abnormal gas−liquid level (**Figure 2A**), and computed tomographic (CT) scan showed apparent bowel wall thickening and intestinal stenosis (**Figure 2B**). The patient underwent surgery to remove the small intestinal stenosis due to the ineffectiveness of medicinal treatments at the age of 26 months. At laparotomy, gross examination showed that the proximal small intestine was dilated, and the distal small intestinal wall was markedly thickened and edematous (**Figure 3A**). One ileal stenotic region of 20 cm was surgically removed (**Figure 3B**). The resected ileal tissue was stiff, and one stenosis with mucosal ulcerations was observed (**Figures 3B, C**). Pathological examination of the lesion location showed that the intestinal mucosa was substituted with granulation tissue with neutrophilic infiltration, and no crypts or epithelial cells were seen (**Figure 4**). Viral tests for cytomegalovirus and Epstein–Barr virus were all negative. Finally, the patient was diagnosed with GI-GvHD complicated with small bowel obstruction. An enteral diet was gradually given after flatus and bowel movement recovery. The patient significantly improved and was discharged after 57 days of hospitalization. The patient received steroids as maintenance treatment after discharge. The timeline of the treatments is summarized in **Figure 1**. As of this writing, the patient is 42 months old, her condition remains stable, and she weighs 13 kg (P14). The patient is monitored closely to be aware of symptoms recurrence and the development of chronic GvHD.

## Discussion

GvHD is a systemic disorder caused by immunological rejection of host tissues by graft immune cells. It remains a major challenge after HCT due to its high morbidity and mortality. GvHD can affect multiple host organs, and the GI is one of the most frequently involved systems (3). GI-GvHD complicated with hemorrhage, perforation, or acute or chronic obstruction occurs in 20%–30% of all patients under HCT (6, 9). The common clinical manifestations of GI-GvHD include diarrhea, abdominal pain, nausea, vomiting, mucositis, and mucosal ulceration (10). Intestinal hemorrhage, perforation, and stricture with bowel obstruction can also occur in severe GI-GvHD (6, 7, 10), but they are rare. In this report, we describe a case of GI-GvHD following HCT for AML in a young child from China. Our patient suffered from watery diarrhea, which progressed to bloody diarrhea after 40 days of HCT. Although treatment with cyclosporine, basiliximab, and methylprednisolone allowed a transient improvement of the diarrhea, the patient experienced prolonged and repeated mucous or bloody stool after the withdrawal of cyclosporine and the gradual reduction in methylprednisolone. Plain abdominal radiography and CT scans showed apparent bowel wall thickening and intestinal stenosis 10 months after HCT. Finally, the patient underwent surgery to remove the small intestinal stenosis due to the ineffectiveness of internal medicinal treatments at the age of 26 months. The patient fully recovered, helped by appropriate medical therapies and nutritional support, after 57 days of hospitalization. She remained well and had no recurrence of GI symptoms as of 28 months after HCT and 16 months after surgery. The exact pathogenesis of GI-GvHD is still unclear. A recent study showed that high glucagon-like peptide-1 (GLP-1) levels in the early posttransplant phase reduced the risk of GI-GvHD by influencing the regeneration of injured epithelial barriers and ameliorating inflammatory responses (11). Furthermore, gut microbiota dysbiosis is associated with GI-GvHD. Burgos da Silva et al. showed that the preservation of fecal microbiome was correlated with reduced severity of GvHD (12).

Timely diagnosis and appropriate treatments are critical in the management of acute GvHD. The primary treatment should not be delayed in patients without classical constellation of symptoms. Biopsies may be helpful for the patients with unclear diagnosis. For patients with steroid-refractory acute GvHD, second-line treatments, such as extra-corporeal photopheresis, anti-tumor necrosis factor antibodies (infliximab, etanercept), mammalian target of rapamycin inhibitors (sirolimus), mycophenolate mofetil, and interleukin-2 receptor antibodies (inolimomab, basiliximab), are needed to be considered and provided appropriately (13). Furthermore, alemtuzumab, pentostatin, mesenchymal stem cells, and methotrexate are suggested as third-line treatment options (13, 14). The patient not receiving timely second-line treatments was critical, as she was suspected with repeated intestinal infections after HCT. Transplant physicians should pay full attention to the management of patients without classical symptoms and steroid-refractory GvHD. The small bowel obstruction of our patient was most likely a presentation of persistent acute GI-GvHD, according to the Mount Sinai Acute GvHD International Consortium (MAGIC) criteria (15).

GvHD complicated with small bowel obstruction in children is rare (6, 7, 16–21). A systematic review conducted by Gutierrez et al. (6) summarized eight pediatric patients with intestinal GvHD who underwent surgical interventions due to small bowel obstruction. Tordjman et al. (7) presented four pediatric cases of small bowel obstruction after HCT with detailed gross and histological data and their genetic status. It was reported that pediatric patients who developed intestinal obstruction always had a history of severe acute GvHD with common symptoms of vomiting, diarrhea, or abdominal pain. Poor clinical response to immunosuppressive therapies leading to chronic GI-GvHD may be a high-risk factor for the development of intestinal obstruction and occlusion. Plain abdominal radiography and CT scans are important tools to observe intestinal obstructions and stenoses in patients with GI-GvHD. In addition, endoscopy and biopsy are helpful to reveal the GI condition of patients with persistent symptoms of vomiting, diarrhea, or abdominal pain (22). Similar to previously reported patients, our patient was less responsive to immunosuppressive therapies and was complicated by long-term GI-GvHD. Small bowel wall thickening and intestinal stenosis were observed 10 months after HCT. In cases of intensive medical treatment failure, surgery may be necessary to remove intestinal stenoses to improve the patient’s chance of survival. Several pediatric patients with small bowel obstruction related to GI-GvHD have been successfully managed by surgical resection of affected loops (6, 7). Nevertheless, the risks of surgical procedures in patients with GvHD should be evaluated prior to the operation, such as hemorrhage, infections, enterocutaneous fistulae, intestinal stricture, and adhesion. In patients complicated with surgery-related hemorrhage, infection, enterocutaneous fistulae, intestinal stricture, and adhesion, bowel obstruction recurrence is prone to arise (6).

In summary, we report a rare pediatric case of severe GI-GvHD complicated with small intestinal obstruction that was refractory to intensive medical treatments. She has been disease-free for 16 months since the surgical resection of the intestinal stenosis. Surgery can be an optimal treatment for GvHD patients with persistent bowel obstruction.

## Data availability statement

The raw data supporting the conclusions of this article will be made available by the authors, without undue reservation.

## Ethics statement

This study was reviewed and approved by Ethical Review Board of Shanghai Children’s Hospital. Written informed consent to participate in this study was provided by the participants’ legal guardian/next of kin.

## Author contributions

TZ and ZL contributed to the study conception and design. JL, YC, and CL acquired the clinical data. YW and BJ drafted the manuscript. TZ and ZL edited and revised the manuscript. All authors contributed to the article and approved the submitted version.

## Funding

This work was supported by the grants from the National Natural Science Foundation of China (grant number 81870373) and the Natural Science Foundation of Shanghai (grant number 22ZR1451800). The funders had no role in study design, data collection and analysis, decision to publish, or preparation of the manuscript.

## Acknowledgments

The authors thank the family for participating and supporting this study.

## Conflict of interest

The authors declare that the research was conducted in the absence of any commercial or financial relationships that could be construed as a potential conflict of interest.

## Publisher’s note

All claims expressed in this article are solely those of the authors and do not necessarily represent those of their affiliated organizations, or those of the publisher, the editors and the reviewers. Any product that may be evaluated in this article, or claim that may be made by its manufacturer, is not guaranteed or endorsed by the publisher.

## Abbreviations

AML, acute myeloid leukemia; GvHD, graft-versus-host disease; HCT, hematopoietic cell transplantation; GI, gastrointestinal; HLA, human leukocyte antigen; CT, computed tomographic.
